# Fecal Microbiota Signatures Are Associated with Response to Ustekinumab Therapy among Crohn’s Disease Patients

**DOI:** 10.1128/mBio.02120-17

**Published:** 2018-03-13

**Authors:** Matthew K. Doherty, Tao Ding, Charlie Koumpouras, Shannon E. Telesco, Calixte Monast, Anuk Das, Carrie Brodmerkel, Patrick D. Schloss

**Affiliations:** a Department of Microbiology and Immunology, University of Michigan, Ann Arbor, Michigan, USA; b Janssen Pharmaceutical Companies of Johnson & Johnson, Spring House, Pennsylvania, USA; University of Maryland School of Medicine

**Keywords:** IBD, Stelara, biologics, biomarkers, inflammatory bowel disease, machine learning, prediction, remission

## Abstract

The fecal microbiota is a rich source of biomarkers that have previously been shown to be predictive of numerous disease states. Less well studied is the effect of immunomodulatory therapy on the microbiota and its role in response to therapy. This study explored associations between the fecal microbiota and therapeutic response of Crohn’s disease (CD) patients treated with ustekinumab (UST; Stelara) in the phase 2 CERTIFI study. Using stool samples collected over the course of 22 weeks, the composition of these subjects’ fecal bacterial communities was characterized by sequencing the 16S rRNA gene. Subjects in remission could be distinguished from those with active disease 6 weeks after treatment using random forest models trained on subjects’ baseline microbiota and clinical data (area under the curve [AUC] of 0.844, specificity of 0.831, sensitivity of 0.774). The most predictive operational taxonomic units (OTUs) that were ubiquitous among subjects were affiliated with *Faecalibacterium* and *Escherichia* or *Shigella*. The median baseline community diversity in subjects in remission 6 weeks after treatment was 1.7 times higher than that in treated subjects with active disease (*P* = 0.020). Their baseline community structures were also significantly different (*P* = 0.017). Two OTUs affiliated with *Faecalibacterium* (*P* = 0.003) and *Bacteroides* (*P* = 0.022) were significantly more abundant at baseline in subjects who were in remission 6 weeks after treatment than those with active CD. The microbiota diversity of UST-treated clinical responders increased over the 22 weeks of the study, in contrast to nonresponsive subjects (*P* = 0.012). The observed baseline differences in fecal microbiota and changes due to therapeutic response support the potential for the microbiota as a response biomarker.

## IMPORTANCE

CD is a global health concern, with increasing incidence and prevalence, causing large economic and health care impacts. Finding prognostic biomarkers that give clinicians the ability to identify patients more likely to respond to CD treatment at diagnosis will reduce the time subjects receive drugs that are unlikely to be beneficial. OTUs associated with remission after treatment induction, especially *Faecalibacterium*, could be biomarkers for successful UST treatment of anti-tumor necrosis factor alpha (anti-TNF-α) refractory CD patients. More broadly, these results suggest that the fecal microbiota could be a useful noninvasive biomarker for directing or monitoring the treatment of gastrointestinal diseases.

## INTRODUCTION

The microbiome has been correlated with a variety of diseases and has shown promise as a predictive tool for disease outcome for gingivitis (1), cardiovascular disease (2), *Clostridium difficile* infection (3, 4), and colorectal cancer (5, 6). Additionally, the microbiome has been shown to alter the efficacy of vaginal microbicides in African women (7), as well as cardiac drugs (8) and cancer treatments (9, 10) in murine models of disease. These results demonstrate that it is possible to use biomarkers from within the microbiome to predict response to therapeutics. In relation to inflammatory bowel disease (IBD), previous studies have shown that the bacterial gut microbiota correlates with disease severity in new-onset, pediatric Crohn’s disease (CD) patients (11, 12). Additionally, recent studies suggest that the gut microbiota could be used to predict clinical response to treatment in adult patients with IBD, including anti-integrin biologics (13, 14) and treatment of pediatric IBD with anti-tumor necrosis factor alpha (anti-TNF-α) or immunomodulators (15, 16). It remains to be determined, however, whether the composition of the fecal gut microbiota can predict and monitor response to biologic CD therapy directed at other targets, such as interleukin 23 (IL-23). Considering the involvement of the immune system and previous evidence for involvement of the microbiome, we hypothesize that response to anti-IL-23 CD therapy can be predicted using microbiome data.

CD is a global health concern causing large economic and health care impacts (17, 18). The disease is characterized by patches of ulceration and inflammation along the entire gastrointestinal tract, with most cases involving the ileum and colon. Currently, individuals with CD are treated based on disease location and risk of complications using escalating immunosuppressive treatment, and/or surgery, with the goal of achieving and sustaining remission (19, 20). Faster induction of remission following diagnosis reduces the risk of irreversible intestinal damage and disability (20–22). Ideally, clinicians would be able to determine personalized treatment options for CD patients at diagnosis that would result in faster achievement of remission (23). Therefore, recent research has been focused on identifying noninvasive biomarkers to monitor CD severity and predict therapeutic response (24–26).

The precise etiology of CD remains unknown, but host genetics, environmental exposure, and the gut microbiome appear to be involved (17, 27). Individuals with CD have reduced microbial diversity in their guts, compared to healthy individuals, with a lower relative abundance of *Firmicutes* and an increased relative abundance of *Enterobacteriaceae* and *Bacteroides* (11, 28–31). Additionally, genome-wide association studies of individuals with CD identified several susceptibility loci, including loci involved in the IL-23 signaling pathway, which could impact the gut microbiota composition and function (19, 28, 32–35). If the fecal microbiota can be used to monitor disease severity and predict response to specific treatment modalities, then clinicians could use it as a noninvasive tool for prescribing therapies that may result in faster remission (36).

The FDA recently approved ustekinumab (UST; Stelara), a monoclonal antibody directed against the shared p40 subunit of IL-12 and IL-23, for the treatment of CD (20, 37–39). Given the potential impact of IL-23 on the microbiota (32–35), we hypothesized that response to UST could be influenced by differences in subjects’ gut microbiota and that UST treatment may alter the fecal microbiota. The effects of biological treatment of IBD on the microbiota are not yet well described but are hypothesized to be indirect, as these drugs act on host factors (19). We analyzed the fecal microbiota of subjects who participated in a double-blind, placebo-controlled phase 2 clinical trial that demonstrated the safety and efficacy of UST for treating subjects with CD refractory to anti-TNF agents (37). The original study found that UST induction treatment had an increased rate of response as well as increased rates of response and remission with UST maintenance therapy compared to placebo. We quantified the association between the fecal microbiota and disease severity, tested whether clinical responders had a microbiota that was distinct from that of nonresponders, and determined whether the fecal microbiota changed in subjects treated with UST using 16S rRNA gene sequence data from these subjects’ stool samples. Our study demonstrates that these associations may be useful in predicting and monitoring UST treatment outcome and suggest that the fecal microbiota may be a broadly useful source of biomarkers for predicting response to treatment.

## RESULTS

### Study design.

**FIG 1:**
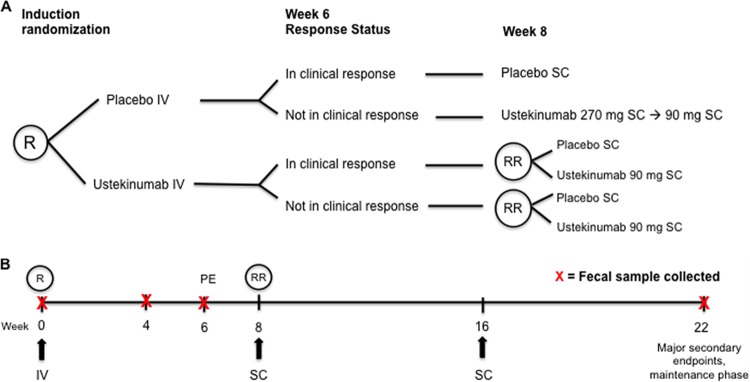
Experimental design as adapted from Sandborn et al. (37). (A) Participants were divided into treatment groups receiving placebo or UST intravenously for therapy. At week 8, subjects were divided into groups receiving either subcutaneous injection of UST or placebo at weeks 8 and 16 as maintenance therapy, based on the response at week 6. Finally, at 22 week, subjects were scored using CDAI for their response to therapy. (B) Stool sampling, treatment, and response evaluation time line. The black arrows indicate treatment administration. Abbreviations: R, randomization; IV, intravenous; PE, primary endpoint; RR, rerandomization (only for subjects receiving UST induction therapy); SC, subcutaneous.

We characterized the fecal microbiota in a subset of anti-TNF-α refractory CD patients, patients with moderate to severe CD, who took part in a randomized, double-blind, placebo-controlled phase 2b clinical trial that demonstrated the efficacy of UST in treating CD (37). Demographic and baseline disease characteristics of this subset are summarized in Table 1. Subjects were randomly assigned to a treatment group in the induction phase of the study and were rerandomized into maintenance therapy groups 8 weeks after induction based on their response (Fig. 1A). In the current study, response was defined as a decrease in a subject’s initial Crohn’s disease activity index (CDAI) of greater than 100 points or remission. Remission was defined as a CDAI below 150 points. The CDAI is the standard instrument for evaluating clinical symptoms and disease activity in CD (40, 41). The CDAI weights patient reported stool frequency, abdominal pain, and general well-being over a week, in combination with weight change, hematocrit, opiate usage for diarrhea, and the presence of abdominal masses or other complications to determine the disease severity score (40, 41). Subjects provided stool samples at baseline (screening) and at 4, 6, and 22 weeks after induction for analysis using 16S rRNA gene sequencing (Fig. 1B). The number of subjects in each treatment group at the primary and secondary endpoints are summarized in Table 2 by their treatment outcome.

**TABLE 1:** Summary of clinical metadata of cohort at baseline^a^

Clinical variable	Value for the following:		
	UST-treated group (*n* = 232)	Placebo-treated group (*n* = 74)	Both groups (*n* = 306)
Age (yr) (mean ± SEM)	38 ± 13	40 ± 14	39 ± 13
Sex (% male)	36.6	43.2	38.2
Race (% Caucasian)	91.8	93.2	92.2
Corticosteroid use (%)	40.1	52.7	43.1
BMI (kg/m^2^) (mean ± SEM)	26 ± 6.7	25 ± 4.9	25 ± 6.3
Disease duration (yr) (mean ± SEM)	12 ± 8.4	13 ± 10	12 ± 8.8
CDAI (mean ± SEM)	330 ± 62	310 ± 69	320 ± 64
Bowel stricture (%)	12.5	10.8	12.1
Tissue involvement (%) (colon/ileocolic/ileal)	28.9/51.3/46	24.3/39.2/36.5	27.8/48.4/23.9

a No significant differences were observed between placebo- and UST-treated groups for any of the listed variables (all *P* = 0.05).

**TABLE 2:** Summary of subjects in each treatment group by endpoint and outcome

Clinical variable (response)	No. of subjects with the indicated response in the following group:	
	UST-treated	Placebo-treated
Wk 6 response (no, yes)	156, 76	48, 26
Wk 6 remission (no, yes)	201, 31	62, 12
Wk 22 response (no, yes)	77, 43	14, 11
Wk 22 remission (no, yes)	96, 24	18, 7

### Association of baseline microbial signatures with treatment remission.

**FIG 2:**
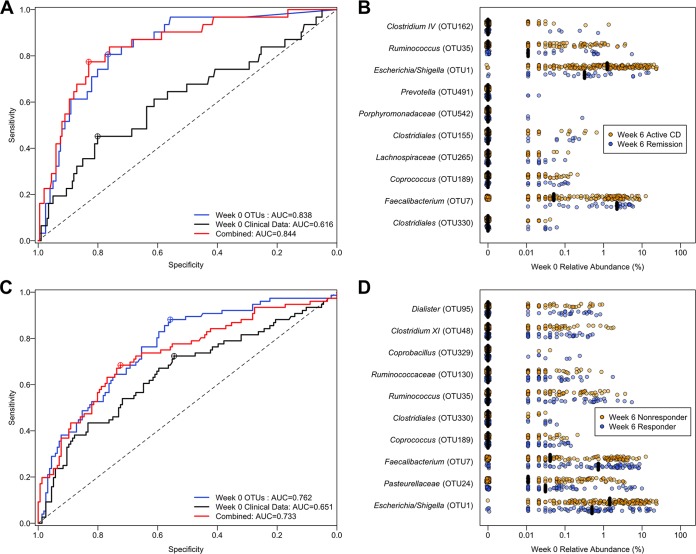
Prediction of week 6 treatment outcome in subjects treated with UST, using baseline samples. (A and C) Receiver operating characteristic (ROC) curves for response (A) and remission (C) using microbiota data (blue), clinical metadata (black), and a combined model (red). (B and D) Top predictive OTUs for the microbiota model based on mean decrease in accuracy (MDA) for response (B) and remission (D). Black bars represent the median relative abundance.

We investigated whether the composition of the baseline fecal microbiota could predict therapeutic remission (CDAI of <150) 6 weeks after induction. To test this hypothesis, we generated random forest (RF) models to predict which subjects would be in remission 6 weeks after induction treatment based on the relative abundance of the fecal microbiota at baseline, clinical metadata at baseline, and the combination of microbiota and clinical data. We determined the optimal model based the largest area under the curve (AUC) of the receiver operating characteristic (ROC) curve for the RF model (6, 42). Clinical data included components of the CDAI, biomarkers for inflammation, and subject metadata described further in Materials and Methods. We trained these models using 232 baseline stool samples from subjects induced with UST; 31 of these subjects achieved remission (Table 2). Clinical data alone resulted in an AUC of 0.616 (specificity of 0.801, sensitivity of 0.452) (Fig. 2A). Using only fecal microbiota data, the model had an AUC of 0.838 (specificity, 0.766; sensitivity, 0.806). Finally, when the clinical metadata and microbiota data were combined, we achieved an AUC of 0.844 (specificity, 0.831; sensitivity, 0.774) for remission 6 weeks after induction. Prediction with clinical metadata alone did not perform as well as models using the baseline fecal microbiome (*P* = 0.001) or the combined model (*P* = 0.001); however, there was not a significant difference between the baseline fecal microbiota model and the combined model (*P* = 0.841).

Optimal predictors were determined based on their mean decrease in accuracy (MDA) in the ability of the model to classify remission from active CD (Fig. 2B). The majority of OTUs identified as optimal predictors in our model for remission had low abundance. However, two OTUs were differentially abundant for subjects in remission 6 weeks after induction treatment. The relative abundance of *Escherichia*/*Shigella* (OTU1) was lower in subjects in remission 6 weeks after induction (median, 1.07%; interquartile range [IQR], 0.033 to 3.70%) compared to subjects with active CD (median, 4.13%; IQR, 0.667 to 15.4%). Also, the relative abundance of *Faecalibacterium* (OTU7) was not only higher in subjects in remission 6 weeks after induction (median, 7.43%; IQR, 1.43 to 11.9%) than subjects with active CD (median, 0.167%; IQR, 0.00 to 5.10%), but it was also present prior to the start of UST treatment in every subject who was in remission 6 weeks after induction.

### Association of baseline microbial signatures with treatment response.

To test whether the composition of the baseline fecal microbiota could predict therapeutic response (CDAI decrease of ≥100 points or remission) 6 weeks after induction, we again used RF models to classify responders from nonresponders 6 weeks after induction (Table 2). Clinical data alone resulted in an AUC of 0.651 (specificity, 0.545; sensitivity, 0.724) (Fig. 2C). Using only microbiota data, the model predicted response with an AUC of 0.762 (specificity, 0.558; sensitivity, 0.882). When clinical metadata and microbiome data were combined, the model predicted response with an AUC of 0.733 (specificity, 0.724; sensitivity, 0.684).

The microbiota model was significantly better able to predict response than the metadata alone (*P* = 0.017), whereas this was not true for the combined model (*P* = 0.069). Additionally, the combined model and the fecal microbiota model were not significantly different in their ability to predict response (*P* = 0.263). Optimal predictors were again determined based on their MDA in the ability of the model to classify response (Fig. 2D). Also, the baseline combined model was significantly better at classifying remission compared to response (*P* = 0.036), whereas this was not true for the fecal microbiota model (*P* = 0.117).

### Comparison of baseline microbiota based on clinical outcome.

As the RF models identified OTUs abundant across this cohort that were important in classification of outcome, we further investigated differences in the baseline microbiota to assess whether they could serve as potential biomarkers for successful UST treatment. We compared the baseline microbiota of all 306 subjects who provided a baseline sample based on treatment group and treatment outcome 6 weeks after treatment induction to assess diversity measures (Table 2). There was no significant difference in diversity based on the responses 6 weeks after induction; however, the baseline *β*-diversity was significantly different by response (*P* = 0.018). No phyla were significantly different by treatment and response (see Fig. S1 in the supplemental material), and no OTUs were significantly different based on UST response or among subjects receiving placebo for induction, regardless of response and remission status.

10.1128/mBio.02120-17.1 FIG S1  Phyla from baseline stool samples in subjects treated with UST by week 6 outcome. (A and B) The relative abundance of each phylum in UST-treated subjects was compared based on response (A) and remission status (B) using a Wilcoxon rank sum test and to identify phyla where there was a *P* value less than 0.05 following a Benjamini-Hochberg correction for multiple comparisons. No comparisons were significant. Whiskers represent the range, and boxes represent the 25 to 75% interquartile range of the median (black bar). Download FIG S1, PDF file, 0.4 MB. Copyright © 2018 Doherty et al. 2018 Doherty et al. This content is distributed under the terms of the Creative Commons Attribution 4.0 International license.

**FIG 3:**
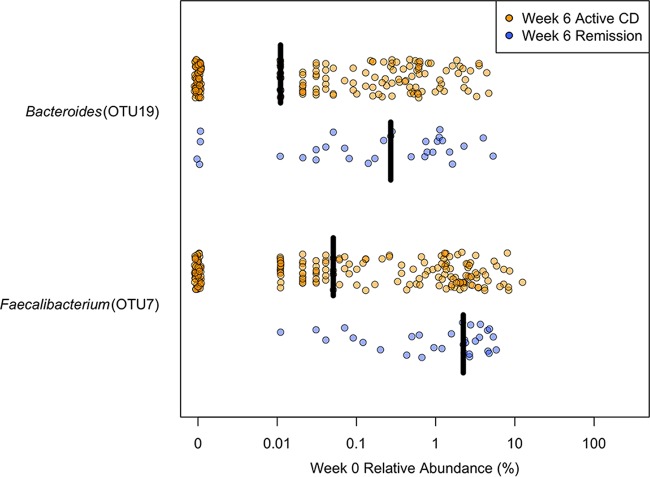
Differential taxa in baseline stool samples from subjects treated with UST, based on week 6 remission status The baseline relative abundance of each OTU was compared between subjects in remission and those with active CD 6 weeks after induction using a Wilcoxon rank sum test followed by a Benjamini-Hochberg correction for multiple comparisons. This identified two OTUs with significantly different relative abundance at baseline (*P* < 0.05). Black bars represent the median relative abundance.

Subjects in remission 6 weeks after induction treatment with UST had significantly higher baseline *α*-diversity based on the inverse Simpson diversity index than subjects with active CD (median values of 11.6 [IQR, 4.84 to 13.4] and 6.95 [IQR, 4.25 to 11.8], respectively; *P* = 0.020). The baseline community structure was also significantly different based on remission status in subjects 6 weeks after induction (*P* = 0.017). Finally, two OTUs were significantly more abundant in subjects in remission 6 weeks after induction compared to subjects with active CD, *Bacteroides* (OTU19) (*P* = 0.022) and *Faecalibacterium* (OTU7) (*P* = 0.003) (Fig. 3).

### Variation in the baseline microbiota is associated with variation in clinical phenotypes.

On the basis of the associations we identified between baseline microbial diversity and response, we hypothesized that there were associations between the microbiota and clinical variables at baseline that could support the use of the microbiota as a noninvasive biomarker for disease activity (36). To test this hypothesis, we compared the baseline microbiota with clinical data at baseline for all 306 samples provided at baseline (Table S1). We observed small but significant correlations for lower *α*-diversity correlating with higher CDAI (*ρ* = −0.161; *P* = 0.014), higher frequency of loose stools per week (*ρ* = −0.193; *P* = 0.003), and longer disease duration (*ρ* = −0.225; *P* = 0.001). Corticosteroid use was associated with 1.45 times higher *α*-diversity (*P* = 0.001). No significant associations were observed between *α*-diversity and C-reactive protein (CRP), fecal calprotectin, or fecal lactoferrin. However, the *β*-diversity was significantly different based on CRP (*P* = 0.033), fecal calprotectin (*P* = 0.006), and fecal lactoferrin (*P* = 0.004). The *β*-diversity was also significantly different based on weekly loose stool frequency (*P* = 0.024), age (*P* = 0.033), the tissue affected (*P* = 0.004), corticosteroid use (*P* = 0.010), and disease duration (*P* = 0.004). No significant differences in *α-* or *β*-diversity were observed for body mass index (BMI), weight, or sex.

10.1128/mBio.02120-17.2 TABLE S1  Diversity differences based on clinical metadata of cohort at baseline. Download TABLE S1, PDF file, 0.2 MB. Copyright © 2018 Doherty et al. 2018 Doherty et al. This content is distributed under the terms of the Creative Commons Attribution 4.0 International license.

### The diversity of the microbiota changes following UST therapy.

**FIG 4:**
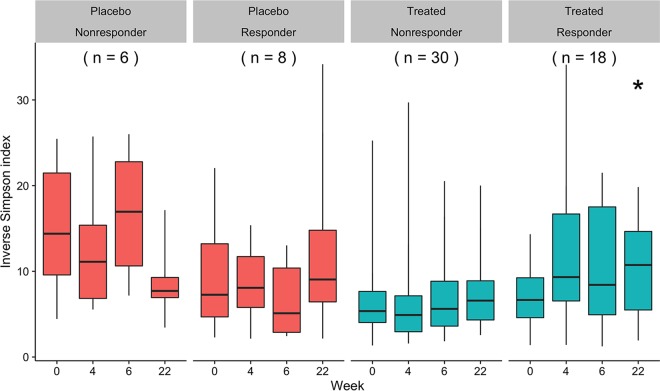
Change in α-diversity over time by induction treatment and week 22 response status. The *α*-diversity of 48 subjects induced and maintained with UST and 14 subjects induced and maintained with placebo was assessed at each time point. Friedman test were performed within each treatment and responder group. Whiskers represent the range and boxes represent the 25 to 75% interquartile range of the median (black bar). The week 22 value is significantly different from the baseline value (*P* < 0.05) as indicated by the asterisk.

We tested whether treatment with UST altered the microbiota by performing a Friedman test comparing *α*-diversity, based on the inverse Simpson diversity index, at each time point within each treatment group based on the subject’s response 22 weeks after therapy. We included 48 subjects induced and maintained with UST (18 responders and 30 nonresponders) and 14 subjects induced and maintained with placebo (8 responders and 6 nonresponders), who provided samples at every time point (Fig. 1). We saw no significant difference in the *α*-diversity over time in subjects who did not respond 22 weeks after induction, regardless of treatment, and in subjects who responded to placebo (Fig. 4). However, the median *α*-diversity of responders 22 weeks after UST induction significantly changed over time (*P* = 0.012) having increased from baseline (median, 6.65; IQR, 4.60 to 9.24) to 4 weeks after UST induction (median, 9.33; IQR, 6.54 to 16.7), decreased from 4 to 6 weeks after induction (median, 8.42; IQR, 4.93 to 17.5), and was significantly higher than baseline (*P* < 0.05) at 22 weeks after induction (median, 10.7; IQR, 5.49 to 14.6).

### The microbiota after UST treatment can distinguish between treatment outcomes.

**FIG 5:**
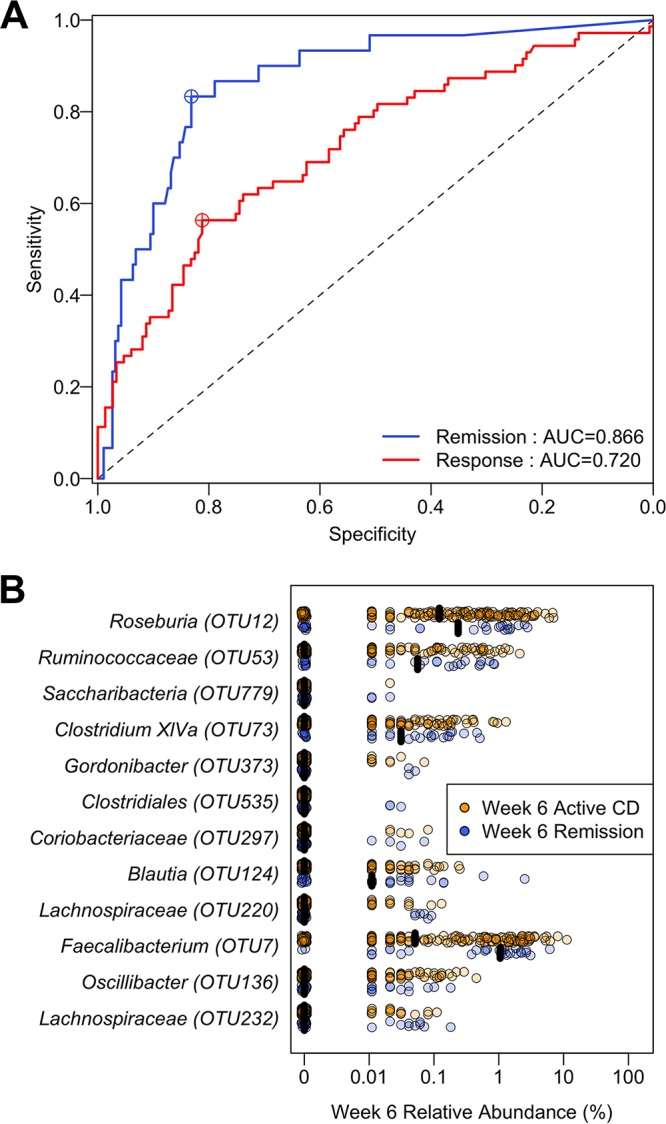
Classification of week 6 response or remission status using week 6 stool samples from subjects treated with UST. (A) ROC curves for week 6 outcome based on the week 6 microbiota. (B) Predictive OTUs from week 6 stool samples for remission status at 6 weeks after induction, based on mean decrease in accuracy. Black bars represent the median relative abundance.

Having demonstrated the microbiome changes in subjects who responded to UST treatment, we hypothesized that the microbiota could be used to monitor response to UST therapy by classifying subjects based on disease activity (36). We again constructed RF classification models to distinguish between subjects by UST treatment outcome based on their fecal microbiota 6 weeks after induction (6, 42). The study design resulted in only 75 stool samples at week 22 from subjects induced and maintained with UST, so we focused our analysis on the 220 stool samples collected at week 6 from subjects induced with UST. We were again better able to distinguish subjects in remission from subjects with active CD than subjects with a clinical response versus no response (*P* = 0.005; Fig. 5A). Our model could classify responses 6 weeks after induction using week 6 stool samples from subjects treated with UST with an AUC of 0.720 (sensitivity, 0.563; specificity, 0.812). For classifying subjects in remission from subjects with active CD 6 weeks after UST induction using week 6 stool samples, the model had an AUC of 0.866 (sensitivity, 0.833; specificity, 0.832). OTUs that were important for these classifications again included *Faecalibacterium* (OTU7), as well as *Blautia* (OTU124), *Clostridium* XIVa (OTU73), *Ruminococcaceae* (OTU53), and *Roseburia* (OTU12). These bacteria were all present at higher median relative abundance in subjects in remission 6 weeks after induction than those with active disease (Fig. 5B).

## DISCUSSION

This study sought to determine whether fecal microbiota can be used to identify patients who will respond to UST therapy and to gain a more detailed understanding of how UST treatment may affect the microbiota. We demonstrated that the microbiota could identify patients more likely to achieve remission following UST therapy than clinical metadata alone in this unique cohort. If this can be validated in future studies with independent cohorts, it may lead to a clinically useful prognostic tool. We also found the fecal microbiota to be associated with CD severity metrics and treatment outcomes. Finally, we found that the microbiota of treated responders changed over time. These results helped further our understanding of the interaction between the human gut microbiota and CD in adult subjects with moderate to severe CD refractory to anti-TNF-α therapies.

The development of predictive models for disease or treatment outcome is anticipated to have a significant impact on clinical decision-making in health care (43). These models may help clinicians decide on the correct course of disease treatment or interventions for disease prevention with their patients. Additionally, patients may benefit with more individualized care that may potentially reduce adverse effects and result in faster recovery, reduce expenses from ineffective therapies, or increase quality of life by preventing disease in high-risk patients.

Our predictive model revealed potential microbial biomarkers indicative of successful UST therapy, which are summarized in Table 3. This allowed us to generate hypotheses about the biology of CD as it relates to the microbiome and UST response. *Faecalibacterium* frequently occurred in our models. It is associated with health, comprising up to 5% of the relative abundance in healthy individuals, and is generally rare in CD patients (28, 30, 44, 45). Each subject in remission 6 weeks after UST therapy had measurable *Faecalibacterium* present at baseline. This supports the hypothesis that *Faecalibacterium* impacts CD pathogenesis. It may even be beneficial to administer *Faecalibacterium* as a probiotic during therapy. *Escherichia/Shigella* also occurred frequently in our models. This OTU is associated with inflammation and has been shown to be associated with CD (45). Many other taxa observed in our analysis had low abundance or were absent in the majority of subjects. However, in many cases, these taxa are related and may serve similar ecologic and metabolic roles in the gut environment. We hypothesize that these microbes may have genes that perform redundant metabolic functions. Performing metagenomics on stool samples in future studies, especially in patients who achieve remission, could reveal these functions, which could be further developed into a clinically useful predictive tool.

**TABLE 3:** Summary of microbial associations with remission at baseline and following UST therapy in UST-treated subjects^a^

Microbial association with remission	Relative abundance at:	
	Baseline	6 wks after UST treatment
*Escherichia/Shigella* (OTU1)	Lower	
*Faecalibacterium* (OTU7)	Higher	Higher
*Roseburia* (OTU12)		Higher
*Bacteroides* (OTU19)	Higher	
*Ruminococcus* (OTU35)	Higher	
*Ruminococcaceae* (OTU53)		Higher
*Clostridium* XlVa (OTU73)		Higher
*Blautia* (OTU124)		Higher

a α-Diversity was higher at baseline.

We were better able to predict whether a subject would achieve clinical remission rather than clinical response, as determined by CDAI score. We hypothesize that this was due to the relative nature of the response criteria compared to the threshold used to determine remission status. While the field appears to be moving away from CDAI and toward patient-reported outcomes and more objectively quantifiable measures such as endoscopic verification of mucosal healing (21, 46), research is ongoing to discover less invasive and more quantifiable biomarkers (24, 25, 36).

We identified several associations between the microbiota and clinical variables that could impact how CD is monitored and treated in the future. Serum CRP, fecal calprotectin, and fecal lactoferrin are widely used as biomarkers to measure inflammation and CD severity. In this study, the microbial community structure was different among subjects based on these markers. These results support the hypothesis that the fecal microbiota could function as a biomarker for measuring disease activity in patients, especially in concert with established inflammatory biomarkers (24, 25, 36). Higher CDAI scores were also associated with lower microbial diversity. This is consistent with other studies on the microbiota in individuals with CD compared to healthy individuals and studies looking at active disease compared to remission (11, 36, 47). However, the CDAI subscore of weekly stool frequency likely drove these differences (see Table S1 in the supplemental material), as we did not observe significant associations between microbial diversity and the other quantitative CDAI subscores. Our observed association between high loose stool frequency and low microbial diversity is consistent with the results of previous studies (48). We also observed differences in the microbial community structure based on disease localization, which is consistent with a study by Naftali et al. (44). Our study also showed that corticosteroid use impacts the composition of the human fecal microbiota, which is consistent with observations in mouse models (49). We also observed that longer disease duration is associated with a reduction in fecal microbial diversity. We hypothesize that prolonged disease duration and the associated inflammation result in the observed decrease in diversity.

Further research into fecal microbiota as a source of biomarkers for predicting therapeutic response could eventually allow for the screening of patients using stool samples at diagnosis to better inform treatment decisions for a wide range of diseases. For CD specifically, using the microbiota to predict response to specific treatment modalities could result in more personalized treatment and faster achievement of remission, thereby increasing patients’ quality of life and reducing economic and health care impacts for CD patients. Our results showing that the *α*-diversity of clinical UST responders increased over time, in contrast to nonresponsive subjects, and our ability to classify subjects in remission from those with active disease following UST treatment are again consistent with other studies suggesting the microbiota could be a useful biomarker for predicting or monitoring response to treatment (36). These predictive biomarkers will need to be validated using independent cohorts in future studies. Additionally, the positive and negative associations between the microbiota and CD allow us to predict the types of mechanisms most likely to alter the microbiota in order to increase the likelihood of achieving a therapeutic response or to monitor disease severity. Prior to the initiation of therapy, patients could have their fecal microbiome analyzed. The microbial community data could then be used to direct the modification of a patient’s microbiota prior to or during treatment with the goal of improving treatment outcomes. Since it has been shown experimentally that the microbiome can alter the efficacy of treatments for a variety of diseases (7–10), if fecal microbiota can be validated as biomarkers to noninvasively predict response to therapy, then patients and clinicians will be able to more rapidly ascertain effective therapies that result in increased patient quality of life.

## MATERIALS AND METHODS

### Study design and sample collection.

Previously, a randomized, double-blind, placebo-controlled phase 2 clinical study of approximately 500 subjects assessed the safety and efficacy of ustekinumab (UST; Stelara) for treating anti-TNF-α refractory, moderate to severe Crohn’s disease (CD) subjects (37) (Fig. 1). Institutional review board approval was acquired at each participating study center, and subjects provided written informed consent (37). Inclusion/exclusion criteria and concomitant medication handling are described in full in the supplemental “Protocol” of the published clinical study (37). Briefly, for inclusion in this study, subjects must have been over the age of 18 years and diagnosed with CD for at least 3 months prior to study initiation, have active CD with a baseline Crohn’s disease activity index (CDAI) score between 220 and 450, and refractory to anti-TNF-α treatment. Subject data were deidentified for our study. Participants provided a stool sample prior to the initiation of the study and were then divided into treatment groups. An additional stool sample was provided 4 weeks after treatment. At 6 weeks after treatment, an additional stool sample was collected, subjects were scored for their response to UST based on CDAI, and then divided into groups receiving either subcutaneous injection of UST or placebo at weeks 8 and 16 as maintenance therapy. A clinical response was defined as a reduction from baseline CDAI score of 100 or more points or as remission in subjects with a baseline CDAI score between 220 and 248 points (37). Remission was defined as a CDAI below the threshold of 150. Finally, at 22 weeks after treatment, subjects provided an additional stool sample and were then scored using CDAI for their response to therapy. Of these samples, 306 were provided prior to treatment, 258 were provided at week 4, 289 at week 6, and 205 at week 22 after treatment, for a total of 1,058 samples. Stool samples were collected by the patients at home, kept refrigerated for no more than 24 h, and then brought to the clinical sites and frozen. Frozen fecal samples were shipped to the University of Michigan and stored at −80^∘^C prior to DNA extraction.

### DNA extraction and 16S rRNA gene sequencing.

Microbial genomic DNA was extracted using the PowerSoil-htp 96-well soil DNA isolation kit (Mo Bio Laboratories) and an EPMotion 5075 pipetting system (5, 6). The V4 region of the 16S rRNA gene from each sample was amplified and sequenced using the Illumina MiSeq platform (24). Sequences were curated as described previously using the mothur software package (v.1.34.4) (50, 51). Briefly, we curated the sequences to reduce sequencing and PCR errors (52), aligned the resulting sequences to the SILVA 16S rRNA sequence database (53), and used UCHIME to remove any chimeric sequences (54). Sequences were clustered into operational taxonomic units (OTUs) at a 97% similarity cutoff using the average neighbor algorithm (55). All sequences were classified using a naive Bayesian classifier trained against the RDP training set (version 14), and OTUs were assigned a classification based on which taxonomy had the majority consensus of sequences within a given OTU (56).

Following sequence curation using the mothur software package (50), we obtained a median of 13,732 sequences per sample (IQR, 7,863 to 21,978). Parallel sequencing of a mock community had an error rate of 0.017%. To limit the effects of uneven sampling, we rarefied the data set to 3,000 sequences per sample. Samples from subjects who completed the clinical trial and for whom we had complete clinical metadata were included in our analysis. Additionally, detailed and reproducible descriptions of how the data were processed and analyzed can be found at https://github.com/SchlossLab/Doherty_CDprediction_mBio_2017.

### Gut microbiota biomarker discovery and statistical analysis.

R v.3.3.2 (31 October 2016) and mothur were used to analyze the data (57). To assess *α*-diversity, the inverse Simpson index was calculated for each sample in the data set. Spearman correlation tests were performed to compare the inverse Simpson index and continuous clinical data. Wilcoxon rank sum tests were performed for pairwise comparisons, and Kruskal-Wallis rank sum tests were performed for comparisons with more than two groups (58, 59). To measure β-diversity, the distance between samples was calculated using the θYC metric, which takes into account the types of bacteria and their abundance to calculate the differences between the communities (60). These distance matrices were assessed for overlap between sets of communities using the nonparametric analysis of molecular variance (AMOVA) test as implemented in the adonis function from the vegan R package (v.2.4.4) (61). Changes in *α*-diversity over time based on week 22 response was assessed using a Friedman test on subjects who provided a sample at each time point (62). The Friedman test is a function in the stats R package (v.3.4.2). Multiple comparisons following a Friedman test were performed using the friedmanmc function in the pgirmess package (v.1.6.7) (63). Changes in *β*-diversity over time by treatment group and response were assessed using the adonis function in vegan stratified by subject. We used the relative abundance of each OTU, *α*-diversity, age, sex, current medications, body mass index (BMI), disease duration, disease location, fecal calprotectin, fecal lactoferrin, C-reactive protein, bowel stricture, and CDAI subscores as input into our random forest (RF) models constructed with the AUCRF R package (v.1.1) (64) to identify phylotypes or clinical variables that distinguish between various treatment and response groups, as well as to predict or determine response outcome (65). Optimal predictors were determined based on their mean decrease in accuracy (MDA) of the model to classify subjects. Differentially abundant OTUs and phyla were selected through comparison of clinical groups using Kruskal-Wallis and Wilcox tests, where appropriate, to identify OTUs/phyla where there was a *P* value less than 0.05 following a Benjamini-Hochberg correction for multiple comparisons (66). Other R packages used in our analysis included ggplot2 v.2.2.1 (67), dplyr v.0.7.4 (68), pROC v.1.10.0 (69), knitr v.1.17 (70), gridExtra v.2.3 (71), devtools v.1.13.3 (72), knitcitations v.1.0.8 (73), scales v.0.5.0 (74), tidyr v.0.7.2 (75), Hmisc v.4.0.3 (76), and cowplot v.0.8.0 (77). A reproducible version of this analysis and manuscript is available at https://github.com/SchlossLab/Doherty_CDprediction_mBio_2017.

### Data availability.

All raw sequence files and a MIMARKS spreadsheet with deidentified clinical metadata have been uploaded into the NCBI Sequence Read Archive (accession no. SRP125127) and are available at https://www.ncbi.nlm.nih.gov/bioproject/PRJNA418765.
