# Supplementary figures and images for: Fecal Microbiota Signatures Are Associated with Response to Ustekinumab Therapy among Crohn’s Disease Patients
Source: mBio. 2018 Mar 13;9(2):e02120-17. doi: 10.1128/mBio.02120-17 (PMC5850325; doi:10.1128/mBio.02120-17)

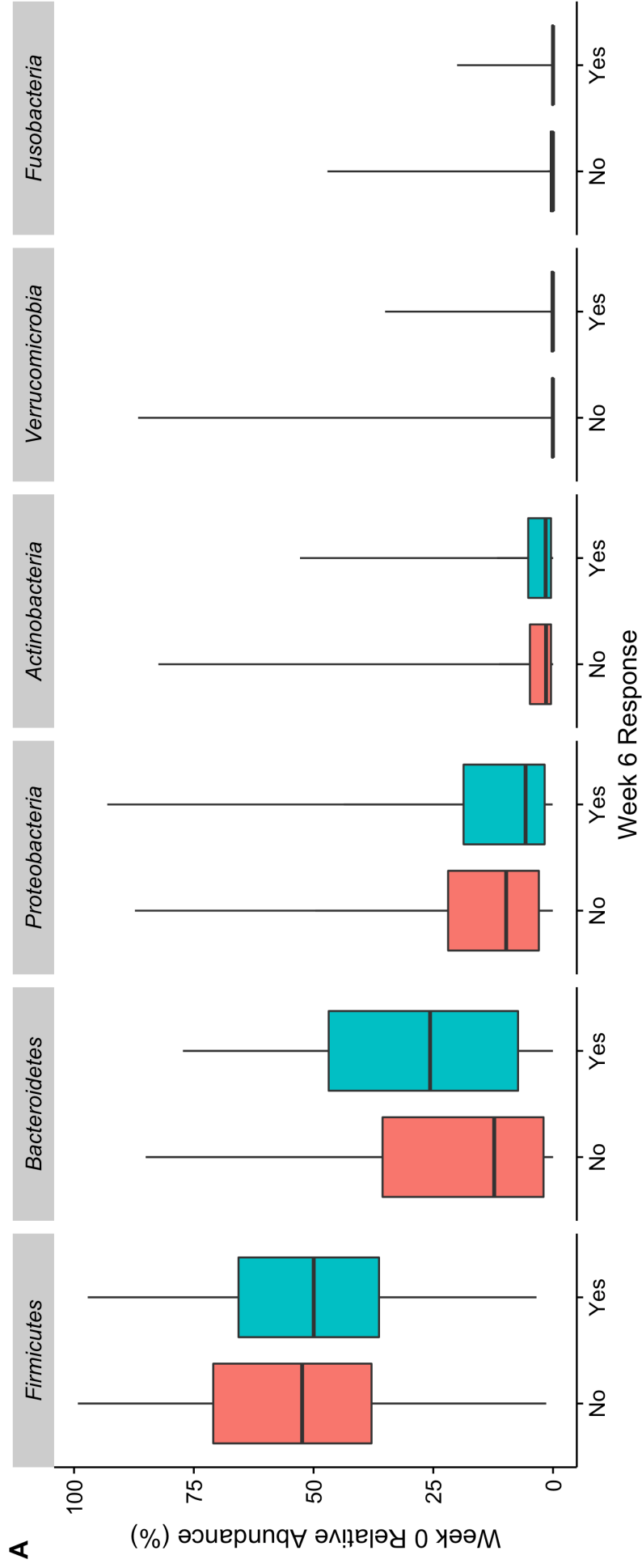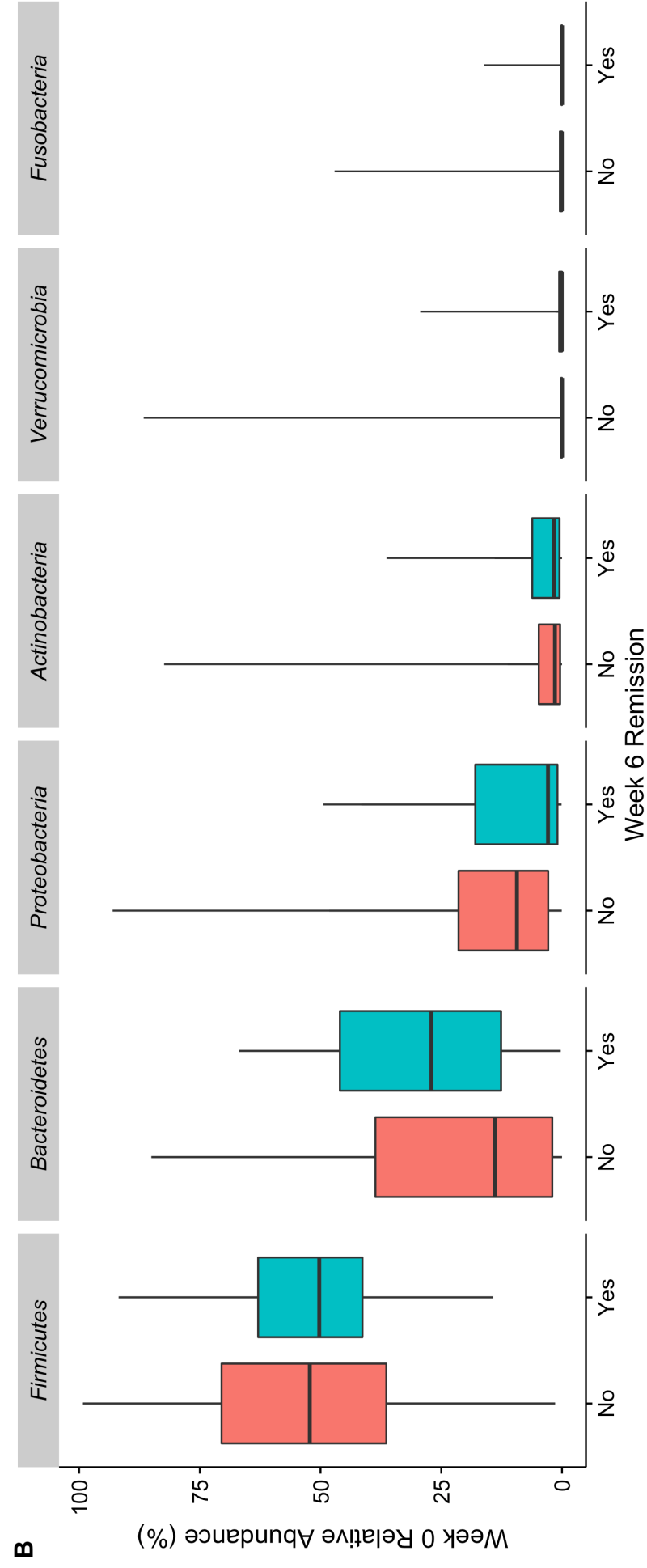

Supplement: FIG S1 [file mbo002183777sf1.pdf]
