# Supplementary material for: Fecal Microbiota Signatures Are Associated with Response to Ustekinumab Therapy among Crohn’s Disease Patients
Source: mBio. 2018 Mar 13;9(2):e02120-17. doi: 10.1128/mBio.02120-17 (PMC5850325; doi:10.1128/mBio.02120-17)
Supplement: TABLE S1 [file mbo002183777st1.pdf]

Supplemental Table 1: Diversity differences based on clinical metadata of cohort at baseline

Clinical Variable	Correlation	Alpha-Diversity (p-value)	Beta-Diversity (p-value)
CDAI	ρ = -0.2	0.014	0.324
Loose Stool Frequency (per week)	ρ = -0.2	0.003	0.024
C-Reactive Protein (mg/L serum)	ρ = 0.06	0.394	0.033
Fecal Calprotectin (μg/g)	ρ = 0.08	0.254	0.006
Fecal Lactoferrin (μg/g)	ρ = 0.1	0.070	0.004
BMI	ρ = 0.07	0.299	0.277
Weight (kg)	ρ = 0.07	0.299	0.112
Age (years)	ρ = -0.05	0.472	0.033
Sex (F/M)	-	0.539	0.277
Corticosteroid Use (Y/N)	-	0.001	0.010
Disease Duration (years)	ρ = -0.2	0.001	0.004
Tissue Involvement	-	0.190	0.004
